# Shifts in the Bacterial Community Related to Quality Properties of Vacuum-Packaged Peeled Potatoes during Storage

**DOI:** 10.3390/foods11081147

**Published:** 2022-04-15

**Authors:** Zudi Li, Wenting Zhao, Yue Ma, Hao Liang, Dan Wang, Xiaoyan Zhao

**Affiliations:** 1 Institute of Agri-Food Processing and Nutrition, Beijing Academy of Agriculture and Forestry Sciences, Beijing Key Laboratory of Agricultural Products of Fruits and Vegetables Preservation and Processing, Key Laboratory of Vegetable Postharvest Processing, Ministry of Agriculture and Rural Affairs, Beijing 100097, China; lizudisyau@163.com (Z.L.); zwtcau@163.com (W.Z.); may_nercv@163.com (Y.M.); wd_nercv@163.com (D.W.); 2 Longda Food Group Co., Ltd., Jinan 265231, China; lh_3000@126.com

**Keywords:** vacuum packaging, peeled potato, bacterial community, quality, spoilage, correlation analysis

## Abstract

To reveal the potential relationship between the bacterial community and quality attributes of vacuum-packaged peeled potatoes, the bacterial community dynamics, visual quality, organic acids, flavor and volatile organic compounds (VOCs) during 12 days of storage under 10 °C were studied, and a correlation analysis was performed between the bacterial community and VOCs. During the whole storage, the dominant bacteria changed from *Ralstonia*, *Pseudomonas*, *Pantoea* and *Comamonas* to *Clostridia*, *Clostridium*, *Lacrimispora*, *Lactococcus* and *Leuconostoc*. The visual quality and hardness deteriorated significantly on day 12; meanwhile, lactic and acetic acid content accumulated to 0.79 and 4.87 mg/g FW, respectively. Potatoes’ flavor deteriorated severely after 8 days, as evidenced by results of an electronic nose (e-nose). A total of 37 VOCs were detected, and the total content showed an increasing trend from 2164.85 to 10658.68 μg/kg during the whole storage. A correlation analysis showed that *Enterobacteriaceae*, *Erwinia*, *Lacrimispora*, *Lactococcus*, *Serratia*, *Pantoea*, *Clostridium*, *Flavobacterium* and *Clostridia* were positively correlated with the biosynthesis of VOCs. In addition, 10 spoilage markers were screened according to a variable importance in projection (VIP) ≥ 1. Ethanol, which was the most abundant spoilage marker, was significantly related to *Enterobacteriaceae*, *Erwinia*, *Lacrimispora* and *Lactococcus*. The results of this study have great practical significance for prolonging the shelf life of fresh-cut agricultural produce.

## 1. Introduction

The fresh-cut fruits and vegetables industry has rapidly grown due to consumers’ increasing demand for convenient foods with a fresh-like quality and high nutritive value. Raw peeled potatoes, as one of the fresh-cut produces that are rich in minerals, dietary fiber and other nutrients, have attracted widespread attention of households and the catering industry [1]. However, potatoes are susceptible to enzymatic browning after peeling, resulting in considerable degradation of sensory quality, safety and market values [2].

Different methods have been studied to prevent the surface browning of potatoes, including blanching, inert gas packaging, low temperatures, radiation treatment, acidification or reduction with antioxidants and the use of chelators or natural extracts [3,4,5]. However, these methods have not been widely applied in practical production due to disadvantages of inconvenient operation, high cost or being unfriendly to environment. Thus, there is an urgent need to study and develop proper techniques to inhibit browning.

Vacuum packaging is one of the most important techniques to prevent browning of fresh-cut fruits and vegetables, such as fresh-cut peaches [6], lotus roots [7,8] and potatoes [9], etc. It has also been reported to maintain better hardness, moisture content, microbial safety and visual quality in comparison with air packaging and other modified atmosphere packaging [9,10,11]. Nevertheless, vacuum packaging has an extremely low oxygen concentration that could promote the growth, fermentation and metabolism of anaerobic pathogens [12,13] and result in sensorial spoilage, particularly an off-odor, of fresh-cut produce [14]. The microbial load and sensorial quality are two main criteria to define the shelf life of fresh-cut fruits and vegetables. Extensive studies [15,16] have reported sensorial quality decay and microbial growth separately in fresh-cut produce, but the potential correlation between particular bacteria and quality decay have rarely been investigated. Some studies have reported the effects of vacuum packaging on soluble solids, enzyme activity and phenol content in fresh-cut potatoes [17]. However, no information has been published concerning the effects of vacuum packaging on the bacterial community, especially on the correlation between particular bacteria and the formation of flavor.

The objectives of this study were (1) to investigate shifts of the bacterial community and quality attributes in vacuum-packaged peeled potatoes during storage; (2) to reveal the potential relationship between quality attributes, especially flavor quality, and the bacterial community. The results of this research could be of great practical importance in developing effective intervention strategies to extend the shelf life of fresh-cut produce.

## 2. Materials and Methods

### 2.1. Plant Material

Fresh potatoes (*Solanum tuberosum* L. cv Netherlands 15) employed in this experiment were harvested in Zaozhuang, Shandong, China (34°80′ N, 117°33′ E). Potatoes with size uniformity, ground color and no diseases or insect pests were selected for experiment. The whole potatoes were cleaned, peeled manually (1–2 mm) and then washed to remove excess starch and other cellular constituents. Immediately after cleaning, potatoes were strained on a draining bucket to reduce the water on the potato surface, and then, the drained potatoes (6 in each bag) were vacuum-sealed using an automatic vacuum-packer (BEIBOO Automation Instrument Co., Ltd., Beijing, China) for 20 s using the EVOH film (91.18 µm film thickness) with an O_2_ permeability of 0.71 cm^3^/m^2^ 24 h 0.1 MPa. During a period of 12 d of storage at 10 °C, samples were analyzed every 4 days.

### 2.2. DNA Extraction and PCR Amplification

The bacterial community’s DNA was extracted from potato samples using the Fast DNA SPIN Kit for soil (Omega Bio-tek, Norcross, GA, USA). The extracts were checked on 1% agarose gel, and their concentration and purity were determined by a NanoDrop 2000 UV-vis spectrophotometer (Thermo Scientific, Wilmington, DC, USA). The hypervariable region 799F_1193R of the bacterial 16S rRNA gene was amplified using an ABI GeneAmp^®^ 9700 PCR thermocycler (ABI, Carlsbad, CA, USA) (primer pairs: 799F (5’-AACMGGATTAGATACCCKG-3’) and 1193R (5’-ACGTCATCCCCACCTTCC-3’)). Specific PCR amplification was as follows: initial denaturation at 95 °C for 3 min; then, denaturation at 95 °C for 27 cycles for 30 s, annealing at 55 °C for 30 s and extension at 72 °C for 45 s and a single extension at 72 °C for 10 min and an end at 4 °C. A same procedure for 13 cycles was performed after the first amplification. The PCR mixtures contained 5 × TransStart FastPfu buffer 4 μL, 2.5 mM dNTPs 2 μL, forward and reverse primers (5 μM) 0.8 μL, *TransStart* FastPfu DNA Polymerase 0.4 μL, template DNA 10 ng and, finally, ddH_2_O up to 20 μL. PCR reactions were performed in triplicate. According to the manufacturer’s instructions, the PCR product was extracted from 2% agarose gel and purified by the AxyPrep DNA Gel Extraction Kit (Axygen Biosciences, Union City, CA, USA) and then quantified by a Quantus™ Fluorometer (Promega, Madison, WI, USA).

### 2.3. Illumina MiSeq Sequencing and Processing of Sequencing Data

Purified amplicons were pooled in equimolar and paired-end sequenced on an Illumina MiSeq PE300 platform/NovaSeq PE250 platform (Illumina, San Diego, CA, USA), according to the standard protocols by Majorbio Bio-Pharm Technology Co. Ltd. (Shanghai, China).

The raw 16S rRNA gene sequencing reads were demultiplexed, quality-filtered by fastp version 0.20.0 [18] and merged by FLASH version 1.2.7 [19] with the following criteria: (a) Over the 50 bp sliding window, 300 bP readings were truncated at any site receiving an average quality score of <20. Truncated readings <50 bP and readings containing ambiguous characters were discarded; (b) Only overlapping sequences longer than 10 bp were assembled; (c) Primers were completely matched, and two nucleotides were allowed to be mismatched, and readings containing ambiguous bases were deleted. Operational taxonomic units (OTUs) with a 97% similarity cutoff [20,21] were clustered using UPARSE version 7.0.1090 (http://drive5.com/uparse/, accessed on 14 April 2022) [20], and chimeric sequences were identified and removed. The taxonomy of each OTU representative sequence was analyzed by RDP Classifier version 2.2 [22] against the 16S rRNA database using a confidence threshold of 70%.

### 2.4. Microbial Growth Analysis

Potatoes (15 g) were put in the sterile bag (S05D, Land Bridge Technology Co., Ltd., Beijing, China) and mixed with 135 mL NaCl solution (0.8%) using a beating homogenizer (BagMixer 400 W, Interscience Lab Inc., Hanover, MA, USA) for 2 min. The homogenized solution (1 mL) was serially diluted at a ratio of 1:10 with an NaCl solution. Next, 1 mL of the diluted suspensions were mixed with plant count agar and incubated at 37 ± 1 °C for 48 h for total aerobic mesophilics, and the total psychrotrophic bacteria were enumerated using a plant count agar by incubating plates at 28 ± 1 °C for 5 days. Mold and yeast counts were performed in a potato dextrose agar by incubation at 28 ± 1 °C for 5 days. Microbial counts were expressed as log CFU/g of tissue.

### 2.5. Visual Quality Assessment

For the surface color of potatoes within 180 min after opening the bag, this was evaluated using a CM-700d portable colorimeter (Konica Minolta, Inc., Tokyo, Japan). The time that browning started was recorded simultaneously. For the color properties (L^*^: brightness, a^*^: red-green color, b^*^: yellow-blue color), 10 potatoes were taken in each storage period, and five points were taken randomly for each potato. The average of the measured values of 50 points in total was taken as the color value.

### 2.6. Texture Analysis

Potatoes were cut into 1 cm^3^ cubes. The hardness of the potato was analyzed with a TA-XT Plus texture analyzer (Stable Micro Systems Ltd., Godalming, UK). A cylinder puncture probe with a diameter of 5 mm (p/5) was pressed down at 5 mm from contact of the samples at a constant speed of 0.50 mm/s. The hardness was determined by the maximum force (N) during pressing. For the texture measurement, 9 individual tubers from each of the storage days were tested.

### 2.7. Determination of pH and Organic Acids

The pH of the potatoes was measured using a pH meter (Metler-Toledo Instruments Co., Ltd., Shanghai, China). A 2 g sample was placed in a centrifugal tube, to which 3 mL of ultra-pure water was then added. Then, it was centrifuged for 15 min at 10,000 rpm after ultrasonic treatment for 30 min in a water bath. The supernatants were filtered through a 0.22 μm polytetrafluoroethylene membrane before being injected. Organic acids (lactic acid and acetic acid) were analyzed using a high-performance liquid chromatography system (Agilent Technologies, Palo Alto, CA, USA) equipped with a quaternary pump, an oven for controlling the column temperature (which was set at 25 °C), a UV-vis detector and a data acquisition system fitted with a XBridge^®^ C18 column (4.6 mm × 250 mm, 5 μm). The mobile phase consisted of a 0.02 M solution of potassium dihydrogen phosphate buffer (pH 2.88) and methanol (98:2, *v*/*v*). The autosampler was adjusted to an injection volume of 20 μL. Isocratic elution was applied to the mobile phase with a flow rate of 0.6 mL/min. A wavelength of 210 nm was selected for quantification. The calibration curves for each compound were constructed using pure standards at different concentrations [23].

### 2.8. E-Nose

The flavor information of the samples was obtained by an e-nose instrument, which was composed of 10 sensors (W1C, W5S, W3C, W6S, W5C, W1S, W1W, W2S, W2W and W3S). A total of 10 g of potato paste was put into 30 mL headspace vials for 6 replicates per sampling day. The conditions of the e-nose system in this study were as follows: sampling time of 200 s, purging time of 100 s and airflow rate of 300 mL/min. All measurements were made at room temperature (25 °C). The average value of response (188–190 s) of each sensor was extracted for analysis.

### 2.9. Determination of VOCs Using Gas Chromatography-Mass Spectrometry (GC-MS)

The analysis of volatile organic compounds (VOCs) in potatoes was performed using headspace solid-phase microextraction (HS–SPME) combined with GC-MS (7890B GC, 5977B MS; Agilent Technologies, Santa Clara, CA, USA). Samples of 1.5 g were accurately weighed into 20 mL glass vials containing 3 mL of saturated salt water, and then, 20 μL 2-methyl-3-heptanone (0.816 × 10^−2^ μg/μL) was added to each vial as an internal standard. The vials were immediately placed in a heating block to equilibrate for 5 min at 40 °C, and then, a CAR/DVB/ PDMS SPME fiber (50/30 μm) (Stableflex™, Santa Clara, CA, USA) was exposed to the headspace for 30 min at 40 °C. Volatiles were desorbed from the SPME fiber at 250 °C for 5 min in the injector in splitless mode, and helium was used as the carrier gas at a flow rate of 1.0 mL/min. A DB-5 MS column (30 m × 0.25 mm, 0.25 μm film thickness; Agilent, Santa Clara, CA, USA) was used, and the column temperature was set at 40 °C (held for 3 min), increased to 150 °C at 5 °C/min and then increased to 250 °C at 10 °C/min (held for 10 min). MS was performed with an ion source temperature of 230 °C, a mass range of *m*/*z* 30–500 and an electron ionization energy of 70 eV.

### 2.10. Statistical Analysis 

The data were analyzed by an analysis of variance (ANOVA) and Duncan’s test using the statistical products and services solution (version 17.0; SPSS in Chicago, IL, USA) with a significant level at *p* < 0.05. Results were expressed as the mean ± standard deviation. PLS-DA was performed using the SIMCA-P software (version 11.5; Umetrics, Umeå, Sweden). The Originpro 8 software (OriginLab Corporation, Northampton, MA, USA) was used for data plotting.

## 3. Results and Discussion

### 3.1. Bacterial Community

**Figure 1:**
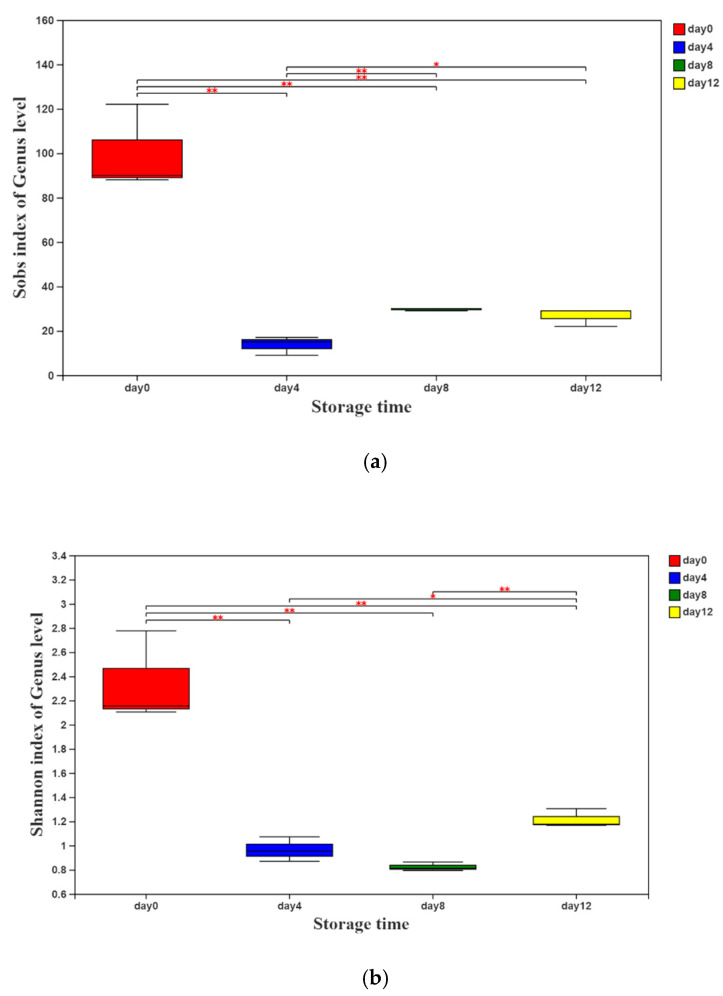
Sobs (**a**) and Shannon (**b**) indices of the genus level of vacuum-packaged peeled potatoes during storage. The significant difference between the storage days, *p* ≤ 0.05, is marked as *, and *p* ≤ 0.01 is marked as **.

According to the sequencing results, the values of the α-diversity indices (Sobs and Shannon indices) are presented in Figure 1a,b. Both of the indices decreased sharply on day 4 and then increased slightly with the extension of storage time, indicating that the richness and diversity of the bacterial community in vacuum-packaged peeled potatoes decreased rapidly first and then increased gradually.

**Figure 2:**
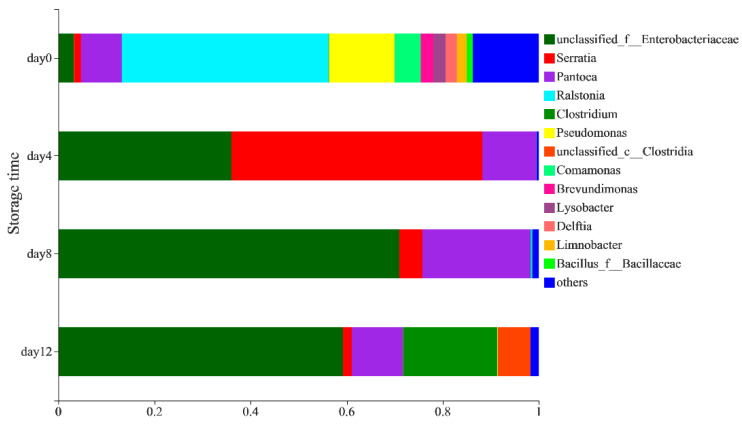
The distribution of bacteria on a genus-level during the storage of vacuum-packaged peeled potatoes.

The distribution of the bacterial community at the genus level (Figure 2) showed that the bacterial community in peeled potatoes was mainly composed of *Ralstonia* (43.08%), *Pseudomonas* (13.66%), *Pantoea* (8.48%), *Comamonas* (5.37%), *Enterobacteriaceae* (3.24%), *Brevundimonas* (2.63%), *Lysobacter* (2.59%), *Delftia* (2.31%), *Limnobacter* (2.1%), *Serratia* (1.43%), *Bacillus_f_Bacillaceae* (1.2%) and others (13.77%) on day 0. The composition and abundance of the bacterial community in vacuum-packaged potatoes changed significantly during storage. A higher relative abundance of *Serratia* (52.27%), *Pantoea* (11.19%), *Erwinia* (0.4%) and *Leuconostoc* (0.03%) was observed in the samples on the 4th day, while the abundance of *Ralstonia*, *Pseudomonas*, *Comamonas* and other genera decreased (Supplementary Table S1). Paillart et al. [24] also found that the relative abundance of *Leuconostoc* spp. Increased. whereas *Pseudomonas* spp. decreased in fresh-cut lettuce during storage, which may have been due to the reduction of oxygen inside the package. On the 8th day, the abundance of *Serratia* (4.86%) decreased significantly, whereas the abundance of *Enterobacteriaceae* (70.95%) and *Pantoea* (22.51%) increased. On the 12th day, the presence of anaerobic bacteria such as *Clostridia* (6.81%), *Clostridium* (19.64%) and *Lacrimispora* (0.38%) (Supplementary Table S1) increased, which could have been due to the development of near-zero oxygen conditions [25]. Avci et al. [26] reported that *Clostridium* strains have the ability to produce ethanol through the fermentation of carbohydrate-rich sources, such as potatoes. *Lactococcus* and *Leuconostoc* were also detected on the 12th day (Supplementary Table S1), which had previously been observed in vacuum-packaged peeled potatoes by Lauridsen et al. [27] and may result in sensorial quality loss by producing organic acids in fresh-cut produce [24,28].

### 3.2. Microbial Quality

**Figure 3:**
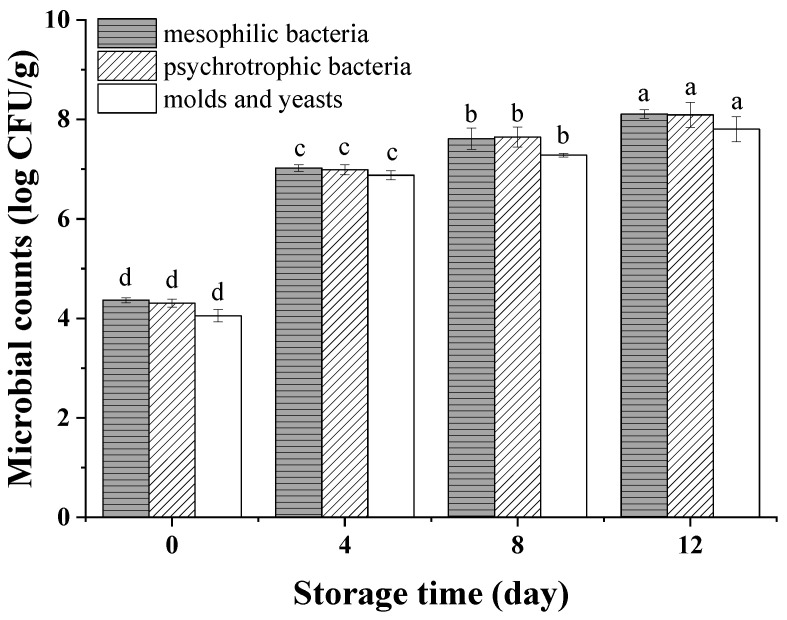
Mesophilic bacteria, psychrotrophic bacteria and molds and yeasts of vacuum-packaged peeled potatoes during storage. The different letters in mesophilic, psychrophilic bacteria and molds and yeasts indicate significant differences (*p* < 0.05).

The initial counts of mesophilic, psychrophilic microorganisms and molds and yeasts on peeled potatoes were 4.36, 4.30 and 4.05 log CFU/g, respectively (Figure 3), which were similar to results of Oms-Oliu et al. [29], who found that these values were 4.09, 3.83 and 3.00 log CFU/g on fresh-cut mushrooms on day 0. The microbial counts increased rapidly from 0 to 4 days, and then, the growth slowed down during the subsequent storage. Waimaleongora-Ek et al. [30] also found a similar trend in fresh-cut sweet potatoes. This trend may have been due to the stable phase of microbial growth or the inhibition of some aerobic microbes by vacuum packaging [31]. Besides this, the slowing-down of microbial growth during the late storage period may also be caused by the increasing of organic acid. [32]. On the 8th day, the count of mesophilic bacteria increased to 7.61 log CFU/g, which is lower than the maximum acceptable contamination value (7.70 log CFU/g) required by the Applied and Environmental Microbiology of France for minimally processed products [33], indicating the potatoes maintained a qualified microbiology load until the 8th day. Moreover, the count of psychrophilic bacteria reached 7.64 log CFU/g, which is lower than 8 log CFU/g, which was reported to be insufficient to cause visual defects of minimally processed vegetables [34,35].

### 3.3. pH and Organic Acids

Changes in the pH and organic acids of vacuum-packaged peeled potatoes are shown in Table 1. The pH of potatoes continuously decreased from 5.91 to 5.60 during storage. The initial contents of lactic acid and acetic acid in raw potatoes were 0.04 and 2.06 mg/g FW, respectively, and they increased to 0.79 and 4.87 mg/g FW on the 12th day. Paillart et al. [28] also found that lactic acid and acetic acid accumulated significantly in fresh-cut lettuce during storage under modified atmosphere packaging, which was attributed to anaerobic fermentation caused by microorganisms. The production of lactic and acetic acid has been reported to be related to the microbial profile and metabolic pattern, and the occurrence of *Leuconostoc* and *Lactococcus* is likely to be the main reason for their production under anaerobic conditions [10,16,36,37]. Paillart et al. [24] found that organic acids, produced by lactic acid bacteria, were not only responsible for a sour off-odor, but also led to loss of membrane integrity, indicating that *Leuconostoc* and *Lactococcus* were correlated to the changes of flavor and texture.

### 3.4. Visual Quality and Hardness

**Figure 4:**
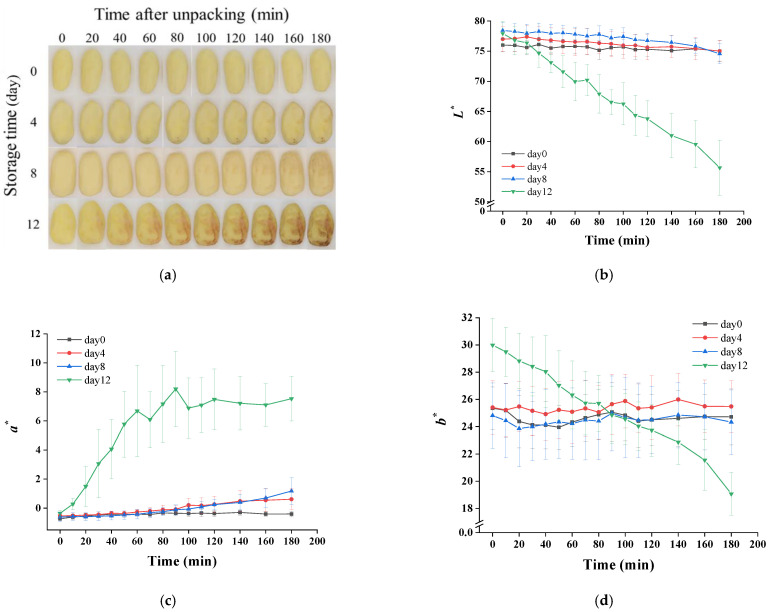
Changes of visual quality (**a**) and the values of L^*^ (**b**), a^*^ (**c**) and b^*^ (**d**) of vacuum-packaged peeled potatoes during storage within 180 min after unpacking.

The color changes of potatoes after unpacking are shown in Figure 4. As exhibited in Figure 4a, a small part surfaces of samples from day 0 to day 8 changed gradually from yellow to brown, whereas a large surface area of the day 12 sample turned pink quickly and then dark brown. The starting time of discoloration for these samples is recorded in Table 1. The surface discoloration of the 0-, 4-, 8- and 12-day samples started at about 31.88, 35.50, 20.35 and 5.71 min, respectively. A delay of browning on day 4 was observed compared to day 0, which could have been due to the inhibition of phenylalanine ammonia lyase activity by the increase of organic acid [38]. The discoloration of day 12 samples happened significantly earlier than other samples, which was also found in the results of the color change measured by the colorimeter (Figure 4b–d). Lightness (L^*^), reddish–greenish (a^*^) and yellowish–bluish (b^*^) values also suggested a browning process of potatoes after opening the package. The samples of day 12 showed an obviously different trend compared to others. As presented in Figure 4b,d, a continual decrease was observed in the L^*^ and b^*^ values. In terms of a^*^ values, day 12 showed an increasing trend firstly, and then it kept constant after 60 min. The aggravation of color change on the 12th day may have been due to cell damage, resulting in a mixing of enzymes with phenolic compounds, which was caused by microbial excessive proliferation and its metabolites’ production [28,38]. In addition, anaerobic bacteria (*Clostridium*) that multiplied during storage in this study have been reported to cause potato slimy rot, manifested by formation of pink pigments (aromatic polyketides metabolites), which can enable the anaerobic bacteria to survive when exposed to an oxygen-rich environment [39]. This could also explain why the potato turned pink rapidly after unpacking on day 12 compared with other samples.

The hardness of potatoes did not change significantly during the first 8 days (Table 1). As the storage was prolonged to 12 days, the potatoes softened obviously in comparison with other samples. The softening of potato tissues could have been due to the accumulation of organic acids, causing an increase of water-soluble pectin [28] or an elevation of pectin-degrading enzymes’ activities [40,41] and then resulting in pectin degradation in the cell wall. Additionally, pectin-decomposing bacteria (*Erwinia*, *Clostridia*) can secrete pectin-degrading enzymes and lead to pectin degradation [39,42].

### 3.5. E-Nose

**Figure 5:**
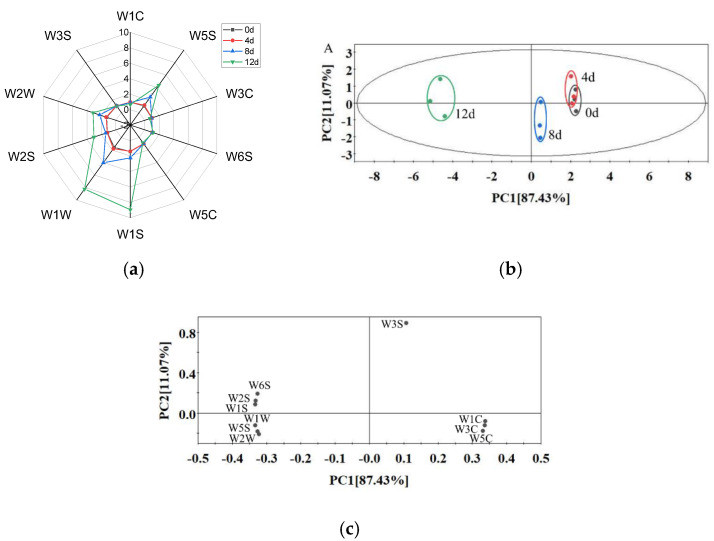
Radar chart (**a**) and PCA score plot (**b**) and loading plot (**c**) from the E-nose data of vacuum-packaged peeled potato samples during storage.

As shown in Figure 5a, the sensor response curve of the sample on day 4 almost overlapped with that of day 0, indicating that potatoes maintained a fresh-like flavor during the first 4 days. With the extension of storage time, the values of the W5S (highly sensitive to nitrogen oxides, furans), W1S (sensitive to hydrocarbons), W1W (sensitive to sulfides and pyrazine), W2S (sensitive to alcohols, aldehydes and ketones) and W2W (sensitive to organic sulfides) sensors increased significantly, which indicates that various compounds, such as nitrogen oxides, furans, sulfides, alcohols and aldehydes, were formed during storage. Chen et al. [43] also found that alcohols, aldehydes and sulfides increased during the storage of fresh-cut bell pepper. 

A principal component analysis (PCA) was used to analyze the differences of the flavor profiles of samples from different storage periods (Figure 5b). PC1 and PC2 explained 87.43% and 11.07% of the total variance, respectively. The cumulative variance contribution rate of the first two principal components reached 98.5%, which explained a large fraction of the overall variability. As the storage period was prolonged, the distance of day 4–12 from day 0 became longer. The PCA profile of day 4 almost overlapped with day 0, while those of day 8 and day 12 were clearly separated from day 0–4, indicating that the flavor quality deteriorated significantly after 8 days of storage and that the e-nose could be used to discriminate the freshness of peeled potatoes. Furthermore, the off-odor may be caused by spoilage bacteria (*Enterobacteriaceae*, *Erwinia*, *Leuconostoc* and *Lactococcus*) due to fermenting sugar [24,28,44].

The loading plot (Figure 5c) showed a relationship among the variables of the e-nose sensors. The loading vectors of W3S, W1C, W3C and W5C had positive scores, and the other loading vectors had negative scores in the PC1 direction. The sensors of W3S, W6S, W2S and W1S had positive scores, and the remaining sensors had negative scores in the PC2 direction. All sensors were far from the origin, indicating that the response values of all sensors had effects on this PCA [45].

### 3.6. VOCs

Volatile organic compounds (VOCs) are closely related to the flavor quality of fresh-cut products, which greatly affects the consumers’ sensory evaluation. A total of 37 VOCs were detected in peeled potatoes during storage period, and they could be categorized as alcohols, aldehydes, ketones, esters, furans and hydrocarbons (Table 2), which was similar to the results of Dresow et al. [46]. Among these VOCs, alcohols, aldehydes and hydrocarbons, which accounted for the major proportion, showed significant upward trends and reached 5304.19 μg/kg, 3638.29 μg/kg and 1415.60 μg/kg at the end of storage, respectively. It is worth noting that esters were only detected in peeled potatoes on day 0, and the content was 17.92 μg/kg. Losses of esters, as an early response to the loss of freshness, have also been reported in fresh-cut cantaloupe [47,48]. In addition, some VOCs (ethanol, 1-pentanol, 3-methyl-1-butanol, 2-methyl-1-butanol, benzyl alcohol, hexanal, (E)-2-hexenal, heptanal, (E)-2-heptenal, (E, E)-2,4-heptadienal, (E)-2-octenal, decanal, 1-penten-3-one, 1-octen-3-one and tetradecane, 2-pentyl-furan) were also found in raw potatoes, as reported by previous studies [46,49,50].

During the whole storage, total content of VOCs exhibited an increasing trend and reached 10,658.68 μg/kg at the end of storage (12 d), which was about 4.9-times that of day 0. This may have been because that spoilage microorganisms produced VOCs under anaerobic conditions by participating in carbohydrate metabolism, amino acid metabolism and other metabolic pathways [41]. Several studies have reported the relationship between microorganisms and the formation of VOCs. For instance, *Erwinia* has the ability to produce short chain alcohols and carbonyl compounds in raw potatoes [51]. Ethanol can originate from the hetero-fermentation of sugar metabolism by *Leuconostoc* [41]. 2-ethyl-1-hexanol may be produced by the action of mold [52]. 3-methyl-1-butanol and 2-methyl-1-butanol, which form off-odors with a fermentative character, are the products of amino acid metabolism under the action of *Pantoea* [35,53,54]. Furthermore, physical damage can result in the mixing of enzymes and nonvolatile precursors in cells to produce VOCs. For example, an intrinsic lipid attacked by enzymes in cut or sliced potatoes could produce a large number of aldehydes and alcohols, such as heptanal, 1-penten-3-one, 1-pentanol, 2,4-heptadienal and 2-pentyl furan [46].

**Figure 6:**
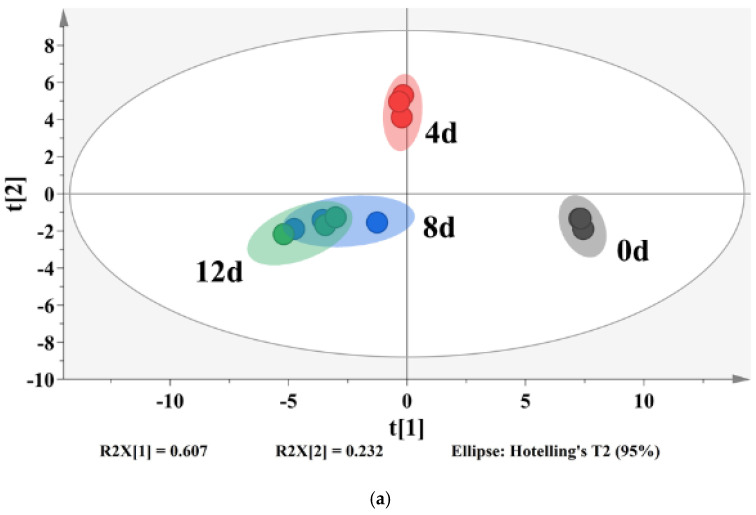

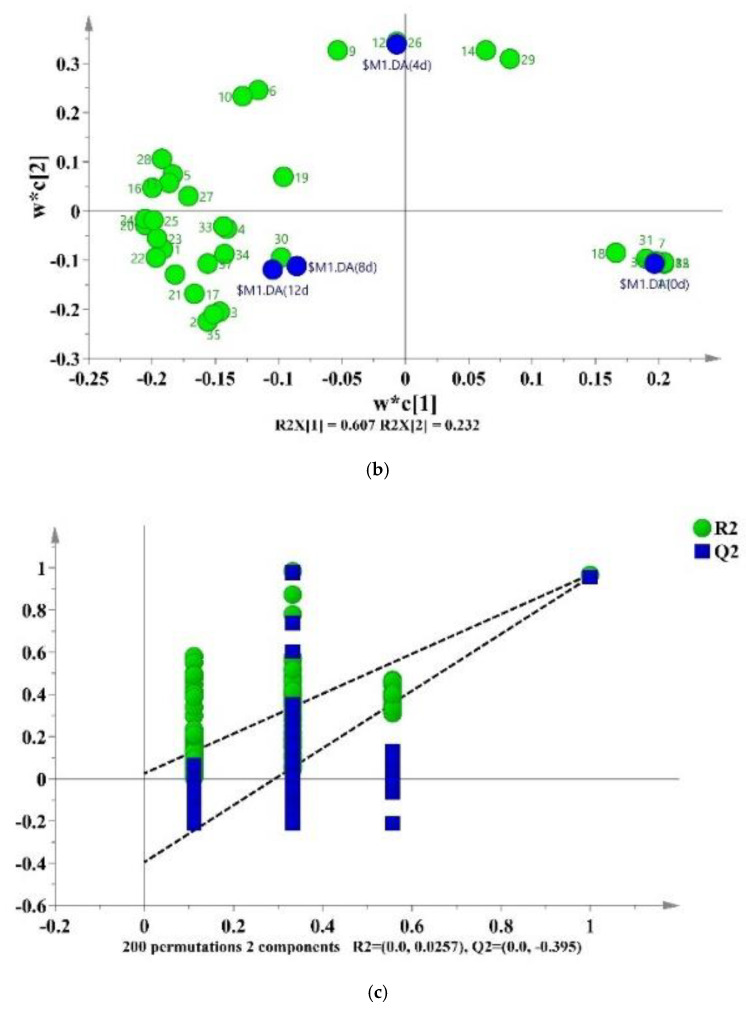
PLS-DA score plot (**a**), loading plot (**b**) and a permutation test at 200 times (**c**) of vacuum-packaged peeled potato samples during storage.

**Figure 7:**
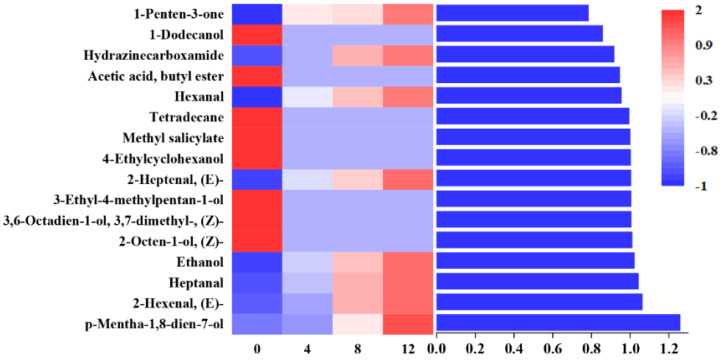
Profile of VOCs with regular changes and values of the VIP of metabolites.

A partial least-squares discriminant analysis (PLS-DA) was used to clarify the effects of storage time on the changes of VOCs in potato samples and to screen for potential spoilage markers. The PLS-DA score plot, loading plot and permutation test at 200 times are displayed in Figure 6. The PLS-DA is a supervised model which can filter system noise and extract variable information. The model fit parameters of R2X, R2Y and Q2 were 0.916, 0.962 and 0.919, respectively, which indicated a good fit and acceptable predictability of the PLS-DA model. Using the PLS-DA model, potato samples in different storage periods were clearly distinguished (Figure 6a). In order to screen for the potential spoilage markers, VOCs related to time evolution were extracted for further analysis (Figure 7). The spoilage markers were defined as VOCs with a variable importance in projection (VIP) ≥ 1. Based on the criterion, *p*-mentha-1,8-dien-7-ol, (E)-2-hexenal, heptanal, ethanol, (Z)-2-octen-1-ol, (Z)-3,7-dimethyl-3,6-octadien-1-ol, 3-ethyl-4-methylpentan-1-ol, (E)-2-heptenal, 4-ethylcyclohexanol and methyl salicylate were selected. Among these compounds, ethanol was the most abundant, with a steady increasing content starting from 1517.1 μg/kg on day 4 and reaching 3843.27 μg/kg on day 12. Moreover, ethanol was related to microbial metabolism and accompanied by the formation of an off-odor, which could lead to consumer rejection [55]. Therefore, ethanol could serve as a better spoilage marker for vacuum-packaged potatoes. Some studies have also treated ethanol as a spoilage marker of fresh-cut produce due to its high concentration [55,56]. Figure 6b shows the corresponding loading plot of potato samples with the VOCs as variables. All VOCs had a relatively important contribution to the discrimination of potatoes in different storage periods. Furthermore, the permutation test demonstrated that the PLS-DA model was reliable, because the slope of both the R2 (0.969) and Q2 (0.957) regression curves were almost near 1 (Figure 6c).

### 3.7. Correlation Analysis between Bacterial Genera and VOCs

**Figure 8:**
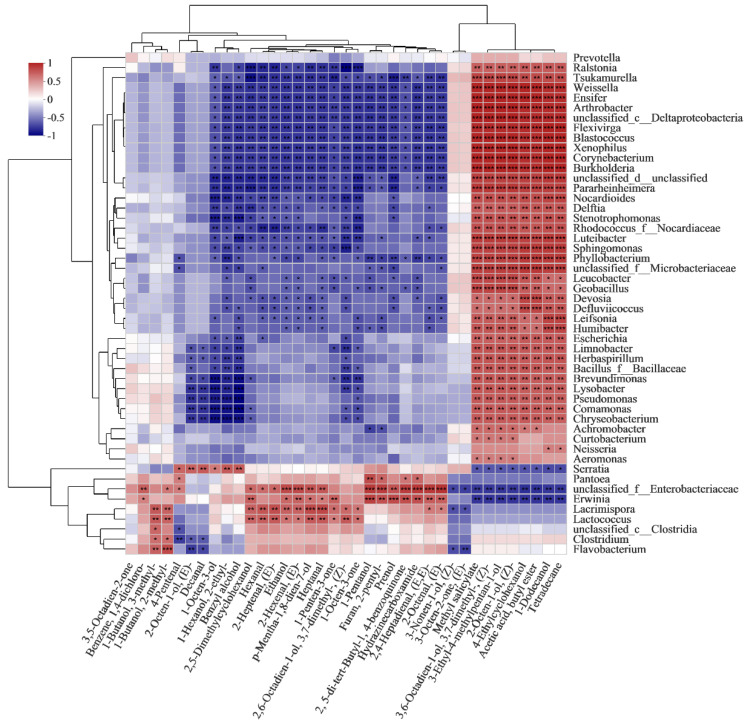
Heatmaps of Spearman correlations between dominant bacterial genera and VOCs during storage.

Among the bacterial genus, the VOCs detected were significantly correlated with 9 bacterial genera. As shown in Figure 8, *Enterobacteriaceae* unclassified was positively correlated with the synthesis of most VOCs, including 17 VOCs (6 alcohols, 7 aldehydes, 1 ketone, 1 furan and 3 hydrocarbons). *Erwinia* was positively correlated with 14 VOCs (5 alcohols, 4 aldehydes, 1 ketone, 1 furan and 3 hydrocarbons). *Lacrimispora* was positively correlated with 14 VOCs (6 alcohols, 6 aldehydes and 2 ketones). *Lactococcus* was positively correlated with 12 VOCs (6 alcohols, 4 aldehydes and 2 ketones). The results suggest that the metabolism of *Enterobacteriaceae*, *Erwinia*, *Lacrimispora* and *Lactococcus* could have a great influence on the changes of VOCs. Rao et al. [57] found that *Enterobacteriaceae* unclassified and *Lactococcus* were dominant in traditional pickled radishes and contributed to the VOCs. *Erwinia* caused red onions stored at 8 °C to produce VOCs of alcohols and aldehydes [58]. With the extension of storage time, the accumulation of alcohols in potatoes, such as the formation of branched chain and linear alcohols, may be related to amino acid metabolism and fatty acyl esters under the action of bacteria, respectively [58]. Besides this, *Serratia*, *Pantoea*, *Clostridium*, *Flavobacterium* and *Clostridia* were positively correlated with 6, 5, 2, 2 and 1 VOCs, respectively. 1-octen-3-ol, 2-ethyl-1-hexanol, benzyl alcohol, (E)-2-octen-1-ol and 4-pentenal and decanal were positively correlated with *Serratia*. Similarly, 1-octen-3-ol and the isomers of (E)-2-octen-1-ol (2-octen-1-ol) were also found in beef inoculated with *Serratia* [59]. 1-pentanol, 4-pentenal, 2-pentyl-furan, hydrazinecarboxamide and 2,5-di-tert-butyl-1,4-benzoquinone were positively correlated with *Pantoea*, which was also reported to be positively correlated with the synthesis of alcohols [60]. The results indicate that *Serratia* and *Pantoea* also impacted the VOCs. Furthermore, spoilage markers were also closely related to the role of bacteria. Ethanol, p-mentha-1,8-dien-7-ol, (E)-2-octen-1-ol, (E)-2-hexenal, heptanal and (E)-2-heptenal were positively correlated with *Enterobacteriaceae*, *Erwinia*, *Lacrimispora* and *Lactococcus*. Among these, ethanol has been reported to be produced through biosynthesis and bioconversion during the growth of *Lactococcus* and other microorganisms in fresh vegetables [57]. Ioannidis et al. [55] also found that the biosynthesis of ethanol was accompanied by the growth of *Lactococcus* in fresh-cut lettuce during the storage. furthermore, *Enterobacteriaceae* has the ability to ferment cheese to produce ethanol [61]. In addition, 3-methyl-1-butanol and 2-methyl-1-butanol, reported as off-odor components, were positively correlated with *Lacrimispora*, *Lactococcus*, *Clostridia*, *Clostridium* and *Flavobacterium*. Several studies have identified 3-methyl-1-butanol and 2-methyl-1-butanol as being associated with *Lactococcus* in cheese. and they could be derived from the proteolytic activity of the bacteria and leucine catabolism [62,63]. The VOCs detected in this study are complex, and several VOCs (ethanol, hexanal, 3-methyl-1-butanol, 2-methyl-1-butanol and 1-octen-3-ol) may produce an unpleasant flavor. In this research, the combination analysis of the bacterial community dynamics and qualities was applied to fresh-cut products, and the potential impact of the bacterial community on the qualities, especially VOCs, of vacuum-packaged peeled potatoes was predicted. It is necessary to further study the VOCs released by specific microorganisms in potatoes to support the possibility of a comprehensive use of some metabolites as a spoilage marker.

## 4. Conclusions

This study revealed the shifts of bacterial diversity and quality attributes of vacuum-packaged peeled potatoes during storage, as well as their potential correlation. Initially, *Ralstonia*, *Pseudomonas*, *Pantoea* and *Comamonas* were dominant, while *Clostridia*, *Clostridium*, *Lacrimispora, Lactococcus* and *Leuconostoc* became more abundant and dominated the bacterial community with the extension of storage time. The visual quality and hardness deteriorated and the contents of lactic acid and acetic acid increased significantly on the 12th day. The flavor quality of potatoes deteriorated significantly after 8 days, as evidenced by results of the e-nose. A total of 37 VOCs were detected, among which alcohols, aldehydes and hydrocarbons accounted for a large proportion. The correlation analysis showed that the accumulation of VOCs was significantly positively correlated to *Enterobacteriaceae*, *Erwinia*, *Lacrimispora*, *Lactococcus*, *Serratia*, *Pantoea*, *Clostridium*, *Flavobacterium* and *Clostridia*. According to a VIP ≥ 1, 10 spoilage markers were screened. Ethanol, which was the most abundant spoilage marker, was significantly related to *Enterobacteriaceae*, *Erwinia*, *Lacrimispora* and *Lactococcus*. Further research should focus on the effects of specific or mixed bacteria on the quality of fresh-cut fruits and vegetables.

## Figures and Tables

**Table 1:** Changes in pH, lactic acid, acetic acid content, hardness and browning time of vacuum-packaged potatoes during storage.

Storage Time (Day)	pH	Lactic Acid (mg/g FW)	Acetic Acid (mg/g FW)	Hardness (*N*)	Browning Time (min)
0	5.91 ± 0.05 ^a^	0.04 ± 0.01 ^d^	2.06 ± 0.07 ^c^	37.76 ± 5.53 ^a^	31.88 ± 6.31 ^b^
4	5.73 ± 0.04 ^b^	0.24 ± 0.04 ^c^	2.20 ± 0.08 ^c^	37.53 ± 3.62 ^a^	35.50 ± 2.84 ^a^
8	5.67 ± 0.06 ^bc^	0.45 ± 0.03 ^b^	3.39 ± 0.65 ^b^	37.13 ± 3.96 ^a^	20.35 ± 3.14 ^c^
12	5.60 ± 0.08 ^c^	0.79 ± 0.09 ^a^	4.87 ± 0.06 ^a^	31.55 ± 6.70 ^b^	5.71 ± 1.29 ^d^

Different superscripts in the same column indicate significant differences.

**Table 2:** The composition and content of VOCs of vacuum-packaged peeled potatoes during storage.

Group (NO.)	Volatile Compounds ^a^	Retention Index ^b^	The Content of Volatile Organic Compounds in Different Storage Times (μg/kg) ^c^			
			0 Day	4 Day	8 Day	12 Day
Alcohols						
1	Ethanol	-	-	1517.10 ± 382.57	2795.72 ± 757.98	3843.27 ± 1004.55
2	1-Butanol, 3-methyl-	732.64	80.46 ± 3.32	61.94 ± 5.31	190.90 ± 13.3	236.17 ± 22.47
3	1-Butanol, 2-methyl-	736.09	76.96 ± 7.02	60.19 ± 8.35	181.93 ± 32.18	246.07 ± 61.24
4	1-Pentanol	764.38	52.70 ± 1.02	79.57 ± 2.77	124.41 ± 34.94	80.63 ± 8.14
5	Prenol	771.74	29.81 ± 6.57	52.00 ± 2.83	55.88 ± 10.13	51.61 ± 0.23
6	1-Octen-3-ol	979.72	307.61 ± 9.78	500.66 ± 27.33	366.60 ± 48.56	436.94 ± 29.16
7	4-Ethylcyclohexanol	992.22	9.95 ± 1.42	-	-	-
8	3-Ethyl-4-methylpentan-1-ol	1019.97	6.72 ± 0.44	-	-	-
9	1-Hexanol, 2-ethyl-	1027.86	130.56 ± 9.05	303.64 ± 27.78	167.53 ± 13.04	165.62 ± 5.27
10	Benzyl alcohol	1032.39	26.21 ± 9.04	87.59 ± 18.30	54.72 ± 12.72	65.00 ± 4.75
11	2-Octen-1-ol, (Z)-	1066.09	5.55 ± 0.02	-	-	-
12	2-Octen-1-ol, (E)-	1069.78	-	20.19 ± 1.62	-	-
13	2,5-Dimethylcyclohexanol	1093.29	11.12 ± 1.65	37.59 ± 6.66	35.81 ± 11.93	46.57 ± 5.82
14	3-Nonen-1-ol, (Z)-	1152.67	6.01 ± 0.50	18.33 ± 1.03	-	-
15	3,6-Octadien-1-ol, 3,7-dimethyl-, (Z)-	1239.06	4.99 ± 0.31	-	-	-
16	2,6-Octadien-1-ol, 3,7-dimethyl-, (Z)-	1248.75	22.63 ± 0.10	48.16 ± 1.16	45.75 ± 1.34	60.51 ± 2.85
17	p-Mentha-1,8-dien-7-ol	1305.40	2.01 ± 0.43	28.49 ± 1.45	48.57 ± 2.00	71.81 ± 1.93
18	1-Dodecanol	1475.17	45.25 ± 34.70	-	-	-
The total content of alcohols (μg/kg)			841.52 ± 32.81	2815.46 ± 385.70	4067.82 ± 892.95	5304.19 ± 1075.33
Aldehydes						
19	4-Pentenal	751.28	-	19.60 ± 3.48	37.00 ± 3.59	-
20	Hexanal	801.71	165.48 ± 7.30	1505.03 ± 78.26	2070.49 ± 543.93	2632.31 ± 87.91
21	2-Hexenal, (E)-	851.48	-	39.32 ± 12.80	122.19 ± 24.57	165.66 ± 50.46
22	Heptanal	901.47	-	18.13 ± 0.47	39.28 ± 11.57	49.46 ± 3.41
23	2-Heptenal, (E)-	956.01	11.23 ± 0.75	178.43 ± 17.41	269.39 ± 84.64	389.63 ± 52.24
24	2,4-Heptadienal, (E, E)-	995.28	12.61 ± 4.72	126.22 ± 9.29	191.98 ± 48.02	186.38 ± 19.20
25	2-Octenal, (E)-	1057.55	12.65 ± 1.42	152.55 ± 8.74	249.89 ± 73.72	214.86 ± 22.23
26	Decanal	1205.13	-	19.56 ± 1.73	-	-
The total content of aldehydes (μg/kg)			201.97 ± 11.08	2058.83 ± 118.58	2980.22 ± 785.10	3638.29 ± 21.61
Ketones						
27	1-Penten-3-one	683.26	15.15 ± 13.43	47.63 ± 21.40	49.33 ± 4.55	62.38 ± 15.28
28	1-Octen-3-one	976.29	43.33 ± 2.45	109.84 ± 11.14	99.48 ± 13.54	117.10 ± 11.62
29	3-Octen-2-one, (E)-	1037.08	12.95 ± 3.75	30.54 ± 5.59	-	-
30	3,5-Octadien-2-one	1068.29	42.17 ± 1.74	45.09 ± 10.65	71.59 ± 15.95	49.86 ± 15.61
The total content of ketones (μg/kg)			113.60 ± 11.39	233.09 ± 10.67	220.41 ± 28.23	229.35 ± 30.40
Esters						
31	Acetic acid, butyl ester	814.23	8.20 ± 3.51	-	-	-
32	Methyl salicylate	1191.07	9.72 ± 0.91	-	-	-
The total content of esters (μg/kg)			17.92 ± 2.89	-	-	-
Furans						
33	Furan, 2-pentyl-	989.12	37.65 ± 6.46	73.43 ± 6.78	130.56 ± 32.65	71.25 ± 15.11
Hydrocarbons						
34	Hydrazinecarboxamide	-	789.47 ± 1.57	910.03 ± 34.95	1110.74 ± 70.22	1180.93 ± 365.58
35	Benzene, 1,4-dichloro-	1013.07	37.88 ± 1.28	34.76 ± 2.35	74.06 ± 12.30	66.69 ± 5.98
36	Tetradecane	1399.22	6.98 ± 1.99	-	-	-
37	2, 5-di-tert-Butyl-1, 4-benzoquinone	1459.44	117.87 ± 16.29	135.48 ± 26.11	186.16 ± 22.54	167.97 ± 22.58
The total content of hydrocarbons (μg/kg)			952.20 ± 19.19	1080.26 ± 14.40	1370.96 ± 100.07	1415.60 ± 393.82
The total content of VOCs (μg/kg)			2164.85 ± 27.65	6261.08 ± 484.63	8769.97 ± 1768.05	10658.68 ± 1467.09

^a^ VOCs detected by the GC-MS compared with the standard mass spectrum in the NIST 17 library (an MS match index ≥ 80% were listed). ^b^ The retention index of each compound was calculated using a series of n-alkanes. ^c^ Each value is the mean of triplicate biological samples. “-” is not detected.

## Data Availability

No new data were created or analyzed in this study. Data sharing is not applicable to this article.

## Supplementary Materials

The following supporting information can be downloaded at: https://www.mdpi.com/article/10.3390/foods11081147/s1. Table S1: Distribution of the identified OTUs of the bacterial genus during storage of vacuum-packaged peeled potatoes.
