# Supplementary material for: Shifts in the Bacterial Community Related to Quality Properties of Vacuum-Packaged Peeled Potatoes during Storage
Source: Foods. 2022 Apr 15;11(8):1147. doi: 10.3390/foods11081147 (PMC9032337; doi:10.3390/foods11081147)
Supplement: Supplementary file 1 [file foods-11-01147-s001.zip › foods-1671360-supplementary.pdf]

Table S1. Distribution of the identified OTUs of bacterial genus during storage of vacuum-ackaged peeled potato.

Genus	OUTs			
	**0d**	**4d**	**8d**	**12d**
g__unclassified_f__Enterobacteriaceae	1232	13681	26962	22492
g__Serratia	544	19865	1847	713
g__Pantoea	3223	4254	8555	4055
g__Ralstonia	16373	8	57	1
g__Clostridium	32	0	1	7463
g__Pseudomonas	5191	1	33	26
g__unclassified_c__Clostridia	16	0	0	2587
g__Comamonas	2042	0	10	3
g__Brevundimonas	1001	0	5	1
g__Lysobacter	984	0	4	2
g__Delftia	877	0	2	0
g__Limnobacter	799	1	3	1
g__Erwinia	0	142	278	229
g__Chryseobacterium	282	0	130	83
g__Bacillus_f__Bacillaceae	455	0	2	1
g__Escherichia	220	31	74	62
g__Corynebacterium	321	0	0	0
g__Phyllobacterium	312	0	0	0
g__Luteibacter	218	0	0	0
g__Rhodococcus_f__Nocardiaceae	187	0	1	0
g__Sphingomonas	179	0	0	0
g__Leifsonia	160	0	0	0
g__Flexivirga	153	0	0	0
g__Tsukamurella	147	0	0	0
g__Flavobacterium	74	0	11	61
g__Lacrimispora	0	0	1	145
g__Neisseria	134	0	0	0
g__Herbaspirillum	127	0	2	2
g__Burkholderia	126	0	0	0
g__Leucobacter	123	0	0	0
g__Stenotrophomonas	99	0	1	0
g__Weissella	95	0	0	0
g__Arthrobacter	76	0	0	0
g__Nocardioides	72	0	1	0
g__Prevotella	65	0	2	0
g__Xenophilus	66	0	0	0
g__Achromobacter	62	0	0	0
g__Geobacillus	62	0	0	0
g__Ensifer	58	0	0	0
g__unclassified_c__Deltaproteobacteria	57	0	0	0
g__Curtobacterium	53	0	0	0
g__unclassified_d__unclassified	53	0	0	0
g__Devosia	53	0	0	0
g__unclassified_f__Microbacteriaceae	50	0	0	0
g__Humibacter	50	0	0	0
g__Blastococcus	49	0	0	0
g__Pararheinheimera	47	0	1	0
g__Defluviicoccus	48	0	0	0
g__Aeromonas	43	0	1	1
g__Lactococcus	0	0	0	43
g__unclassified_c__Actinobacteria	41	0	0	0
g__Mycobacterium	40	0	0	1
g__unclassified_p__Acidobacteria	40	0	0	0
g__Microbacterium	40	0	0	0
g__Staphylococcus	36	1	1	1
g__unclassified_f__Clostridiales_Family_XIII._Incertae_Sedis	37	0	0	0
g__Terrabacter	34	0	1	0
g__unclassified_f__Sporichthyaceae	33	0	0	0
g__Mesorhizobium	30	0	0	0
g__Cellvibrio	28	0	0	0
g__Glutamicibacter	28	0	0	0

g__Acinetobacter	22	0	3	1
g__unclassified_c__Betaproteobacteria	27	0	0	0
g__Mediterraneibacter	26	0	0	0
g__Lactobacillus	26	0	0	0
g__Solirubrobacter	25	0	0	0
g__Dietzia	25	0	0	0
g__Nitrobacter	25	0	0	0
g__Steroidobacter	24	0	0	0
g__unclassified_o__Bacillales	24	0	0	0
g__Paenibacillus	11	0	2	10
g__Leuconostoc	0	13	0	9
g__Anaeromyxobacter	22	0	0	0
g__Conexibacter	19	0	2	0
g__Streptomyces	21	0	0	0
g__Novosphingobium	20	0	0	0
g__unclassified_c__Alphaproteobacteria	20	0	0	0
g__Uliginosibacterium	20	0	0	0
g__Streptococcus	19	0	0	0
g__Bradyrhizobium	18	0	0	0
g__unclassified_p__Candidatus_Saccharibacteria	16	0	1	0
g__unclassified_f__Kofleriaceae	17	0	0	0
g__Paracoccus_f__Rhodobacteraceae	17	0	0	0
g__Ochrobactrum	15	0	0	0
g__Methylovorus	15	0	0	0
g__Dechloromonas	15	0	0	0
g__Cutibacterium	15	0	0	0
g__Methylobacterium	14	0	0	0
g__Pseudochrobactrum	14	0	0	0
g__unclassified_f__Methylocystaceae	13	0	0	0
g__unclassified_o__Sphingomonadales	13	0	0	0
g__Turicibacter	13	0	0	0
g__Fictibacillus	12	0	0	0
g__Ectothiorhodospira	13	0	0	0
g__Solibacillus	12	0	0	0
g__Brachybacterium	12	0	0	0
g__Caulobacter	11	0	0	0
g__unclassified_f__Thermoactinomycetaceae	10	0	0	0
g__unclassified_p__Proteobacteria	10	0	0	0
g__Hydrogenophilus	10	0	0	0
g__Georgenia	10	0	0	0
g__Bosea_f__Bradyrhizobiaceae	9	0	0	0
g__Fusobacterium	8	0	2	0
g__Nitrospira	9	0	0	0
g__Shimazuella	9	0	0	0
g__Micromonospora	10	0	0	0
g__Agromyces	9	0	0	0
g__Bifidobacterium	9	0	0	0
g__Lentzea	9	0	0	0
g__Acidobacterium	9	0	0	0
g__unclassified_f__Lachnospiraceae	8	0	0	0
g__unclassified_o__Myxococcales	9	0	0	0
g__Propioniciclava	8	0	0	0
g__Nonomuraea	8	0	0	0
g__Isoptericola	8	0	0	0
g__Shewanella	8	0	0	0
g__Paraliobacillus	8	0	0	0
g__Pseudarthrobacter	7	0	0	0
g__Azospirillum	7	0	0	0
g__unclassified_p__Gemmatimonadetes_d__Bacteria	6	0	0	0
g__Thermoactinomyces	6	0	0	0
g__unclassified_p__Bacteroidetes	6	0	0	0
g__Actinotalea	6	0	0	0
g__Perlucidibaca	5	0	0	0
g__unclassified_f__Acidobacteriaceae	5	0	0	0
g__Methylorubrum	6	0	0	0
g__Enterobacter	0	0	0	5
g__unclassified_o__Sphingobacteriales	5	0	0	0
g__Lihuaxuella	4	0	0	0

g__Actinomadura	4	0	0	0
g__unclassified_f__Gemmatimonadaceae	3	0	0	0
g__Janthinobacterium	3	0	0	0
g__Pseudonocardia	3	0	0	0
g__Elizabethkingia	3	0	0	0
g__Rubellimicrobium	3	0	0	0
g__unclassified_f__Hyphomicrobiaceae	2	0	1	0
g__unclassified_f__Rhodospirillaceae	3	0	0	0
g__Actinophytocola	2	0	0	0
g__unclassified_c__Gammaproteobacteria	2	0	0	0
g__Blautia	2	0	0	0
g__Agrobacterium	2	0	0	0
g__Solanum	2	0	0	0
g__Microbulbifer	2	0	0	0
g__Dongia	2	0	0	0
g__Halomonas	2	0	0	0
g__unclassified_c__Chlamydiia	2	0	0	0
g__unclassified_o__Spirochaetales	1	0	0	0
g__Nitrosospira	1	0	0	0
g__unclassified_f__Rhodobiaceae	1	0	0	0
g__Methylomonas	1	0	0	0
g__unclassified_c__Sphingobacteriia	1	0	0	0
g__Simplicispira	0	0	0	1
g__Alkaliflexus	0	0	0	0
g__Peptostreptococcus	0	0	0	0
g__Physalis	0	0	0	0
